# The concrete processing of Chinese action metaphors: an ERP study

**DOI:** 10.3389/fpsyg.2024.1362978

**Published:** 2024-04-04

**Authors:** Yuqing Zhang, Shifa Chen, Yule Peng, Xin Yang

**Affiliations:** College of Foreign Languages, Ocean University of China, Qingdao, China

**Keywords:** Chinese action metaphor, concreteness effect, semantics, embodied cognition, N4005

## Abstract

The present research adopts ERP (Event-Related Potentials) technology to investigate whether there exists a concreteness effect in the processing of Chinese action verbs within metaphorical context. The mean amplitudes of N400 activated by action metaphors were compared with those activated by literal verbs and abstract verbs. The findings indicated that the Met verbs evoked a significantly larger N400 response at frontal brain region compared to the Abs verbs at a time window 200–500 ms, while the Met verbs elicited a notably greater N400 amplitude specifically at the posterior brain region in comparison to the Lit verbs at 300–500 ms time window. These results may be interpreted as indicating that the comprehension of the Met verbs is based on the concrete action semantics.

## Introduction

How semantic information is represented is one of the focuses of psycholinguistic research. Classical cognitive theories claim that concepts are represented amodally as abstract symbols, and there is no relationship between semantic system and sensory-motor system (Pylyshin, 1984; Shallice, 1988). Embodied cognition theory, contrasting with the traditional view, holds that semantic information is rooted in the sensorimotor experience of the body (Glenberg and Kaschak, 2002; Barsalou, 2008), language comprehension relies on the reactivation of relevant sensorimotor experiences (Zwaan and Madden, 2005). However, it is still unclear exactly how the semantic system and sensory-motor system interact. Weak embodiment theories propose that sensorimotor systems are only activated when ideas are directly connected to physical activity. Strong embodiment, on the other hand, gives sensorimotor cortex a sweeping role in language comprehension, even of more abstract concepts (Desai et al., 2011). Action-related language is currently a burgeoning area of research within the embodied semantic approach, aiming to investigate the intricate relationship between sensorimotor and semantic systems (Vukovic et al., 2015; Tian et al., 2020, 2023; Monaco et al., 2023). A series of electrophysiological studies have proved the embodiment of the processing of literal action language by showing the activation of the sensorimotor cortex during language comprehension (Tettamanti et al., 2005; Aziz-Zadeh et al., 2006; D’Ausilio et al., 2009; Raposo et al., 2009; Kolb et al., 2010; Moody and Gennari, 2010; Fargier et al., 2012; Moseley and Pulvermüller, 2014; Gianelli and Dalla Volta, 2015; Vukovic et al., 2015; Johari et al., 2022). For example, the processing of literal action sentence “I grasp a knife” elicits activation in a left fronto-parieto-temporal circuit within the premotor cortex, exhibiting somatotopic organization (Desai et al., 2011). Action verbs, however, have a variety of uses. It is not enough to explain the relationship between sensorimotor system and semantic system only by examining the embodiment of action verb embedded in concrete context. In everyday discourse, concrete action verbs are also often metaphorically used to construct abstract concepts that are far from physical experience (Zheng et al., 2018). It is possible to represent abstract concepts through metaphorical mappings to concrete domains (Desai, 2022). For example, comprehending the linguistic expression “He grasped this idea” needs the retrieval of the conceptual mapping “understanding is grasping.” An intriguing opportunity to examine the interaction between semantic system and sensory-motor system is provided by action metaphor since it permits the comparison between the action verbs used in literal context (e.g., grasp a flower) and the same action verbs used in metaphorical context (e.g., grasp an idea) (Desai et al., 2011, 2013).

Recently, a growing body of studies have investigated the question of the embodiment of action metaphor processing by comparing metaphoric action sentences with non-metaphoric ones, and yielded mixed findings (for an overview, see Khatin-Zadeh et al., 2023). Some of the studies have demonstrated the activation of the sensorimotor systems during action metaphor comprehensions (Desai et al., 2011, 2013; Boulenger et al., 2012; Lauro et al., 2013; Schuil et al., 2013; Fernandino et al., 2016; Martin, 2016; Johari et al., 2021). For instance, Desai et al. (2011) used fMRI to examine the processing mechanism of action metaphors. The experimental materials included literal action (e.g., The daughter grasped the flowers, Lit) sentences, metaphorical action (e.g., The public grasped the idea, Met) sentences, and abstract action (e.g., The public understood the idea, Abs) sentences. They found that the left anterior inferior partial lobule (associated with action planning) was activated by both Lit and Met sentences, and the left superior temporal areas (associated with abstract language comprehension) were activated by both Met and Abs sentences. These results were consistent with the view that the sensorimotor systems are involved in the representation of the metaphorical action sentences. Similar findings were also noticed in the study of Lauro and his colleagues. Using fMRI, Lauro et al. (2013) found motor involvement in the processing of literal and metaphorical sentences, while the idiomatic sentences only showed a trend toward significance. The results proved that the understanding of language may activate the motor areas, but to a different degree based on the concreteness of the contexts in which the action-related verbs appear. In another more recent study, the involvement of the motor system in the understanding of metaphorical action sentences was examined by Johari et al. (2021) using high-definition transcranial direct current stimulation (HD-tDCS). The findings demonstrated that the comprehension of metaphorical action sentences involved the left motor brain. However, inconsistent results were also found in the previous related studies. For instance, Aziz-Zadeh et al. (2006) discovered effector-specific activations in response to visually presented literal action sentences, while no such activations were observed for figurative phrases (e.g., “biting off more than you can chew”). Raposo et al. (2009) found that motor areas were significantly activated when action verbs were presented alone, and to a lesser degree, within literal sentence contexts, but the utilization of these same verbs in metaphorical sentences primarily resulted in activation in language processing regions rather than sensorimotor areas.

The above studies only investigated the static activation of the sensorimotor cortex in the processing of action metaphors from the perspective of neuroanatomy. Then when does the sensory-motor recruitment take place during action metaphor understanding? Understanding the time-course of processing can further clarify the embodied nature of action metaphors. If sensory-motor activation occurs early, it would be interpreted as that concrete bodily experiences play a significant role in understanding abstract meaning (Gallese and Lakoff, 2005), and thus supporting the strong embodiment account. If it is engaged later, then it can be viewed as epiphenomenal (Mahon and Caramazza, 2008), and thus supporting the weak embodiment account. ERPs are an excellent approach for researching sentence processing (Kazmerski et al., 2003). Moreover, ERPs are particularly well-suited for investigating the temporal evolution of processing conflicts between arguments within linguistic phenomena (Ji et al., 2020). The classic N400, a negative ERP component peaking around 400 ms after word onset, is often used to explain the concreteness N400 effect of concrete words (Kutas and Federmeier, 2011). To be more specific, the classic N400 is believed to be associated with the semantic processing of linguistics expression, and the N400 effect found in the processing of concrete words is ascribed to a greater activation of semantic information by concrete words compared to abstract words (West and Holcomb, 2000). However, Barber et al. (2013) observed concreteness N400 effect while maintaining a constant context availability between concrete and abstract words, and they argued that the processing of concrete words involves the engagement of multimodal (sensory-motor) aspects from a widely distributed cortical network, while the processing of abstract words elicits distinct and shallower features. Therefore, according to Barber et al. (2013), the underlying knowledge that is recruited rather than the quantity of semantic information activated accounts for the concreteness effect. Furthermore, in contrast to the classic N400 effect, which disperses over the posterior regions, the concreteness N400 effect demonstrates a frontal distribution (Adorni and Proverbio, 2012; Barber et al., 2013; Lai et al., 2019).

Instead of concrete nouns, there have been also studies examining the concreteness effect of verbs. Using ERPs, Dalla Volta et al. (2014) examined the processing time-course of abstract verbs and concrete action verbs. The experiment materials included verbs associated with actions performed by hands, feet, and mouth (e.g., to knit, to kick, to bite) as opposed to abstract controls (e.g., to infer). The study revealed that concrete action verbs exhibited higher mean amplitudes compared to abstract verbs in the frontal motor region of the brain right hemisphere during the 200–300 ms interval. Furthermore, a concreteness effect was observed in the parietal region of the left hemisphere within the 300–400 ms timeframe. The authors suggested that the concreteness N400 may be attributed to two underlying factors: a sensory-motor recruitment associated with frontal activity and a language-sensitive response linked to parietal activation. In another study, Bardolph and Coulson (2014) conducted a study to investigate the impact of the activated motor system (by asking the participants to move the marbles up and down) on the processing of verbs with varying degrees of spatial attributes both in their literal (e.g., ascend, descend) and metaphorical (e.g., inspire, defeat) senses. The study revealed that for literal verbs, in the time range of 200–300 ms, incongruent condition elicited a larger negativity effect compared to congruent condition. In contrast, for metaphorical verbs, the incongruency effect was observed only after 500 ms from word onset. These findings indicated that a neural response related to direct physical representation was observed between 200 and 300 ms after the presentation of words, whereas a neural response associated with figurative embodiment appeared more than 500 ms after word initiation. However, these investigations solely focused on verbs presented in isolation. In an ERP experiment, Lai et al. (2019) investigated the activation timing of the sensorimotor systems during the processing of action verbs in literal (e.g., The man bent the rod) and metaphorical sentence contexts (e.g., The church bent the rules) based on the concreteness N400 effect. Frontally distributed N400 effects were obtained in the literal-abstract contrast (200–300 ms after verb onset) and the metaphor-abstract contrast (200–500 ms after verb onset), and posterior N400 effect was acquired in the metaphor-concrete contrast (200–400 ms after verb onset). They authors concluded that the literal sense of the verb in the metaphorical context is activated early and maintained throughout the 200–500 ms window.

The present study conducted an ERPs experiment to explore whether there exists a concreteness effect for Chinese action verbs in metaphorical context. Specifically, we employed three types of sentences: literal action sentences (e.g., The doctor broke the cup), abstract sentences (e.g., The company violated the rules) and metaphoric action sentences (e.g., The company broke the rules). One possibility is that the action metaphors are primarily comprehended as their concrete semantics, then the concreteness effect would be found in the processing of action metaphors. An alternative is that the action metaphors are comprehended partly through concrete semantics and partly through further cognitive processes, then the metaphor-abstract contrast and literal-abstract contrast should have different activation timings and topographies. A further alternative is that the action metaphors are comprehended abstractly, then the metaphor-abstract contrast should not exhibit any resemblance to the concreteness effect.

## Methods

### Participants

Participants in the ERPs experiment were 25 healthy Chinese native speakers (9 males and 16 females ranging in age from 19 to 23, mean age 20.98), with no history of linguistic or neurological impairment. Two additional participants were removed due to excessive ERP artifacts. They were all undergraduate students enrolled at Ocean University of China. All the participants were right-handed and had normal or corrected-to-normal vision. Prior to participating in the experiment, informed consent was obtained from each individual. Following the completion of the experiment, participants received financial compensation.

### Materials

The experimental material consisted of 23 triples, including 69 sentences in three conditions of literal action (Lit), metaphorical action (Met), and abstract action (Abs). Twenty-seven Filler sentences were also used. All the experimental sentences took the same syntactic structures (Subject-Predicate-Object) and were constructed in triples consisting of one sentence from each condition (see examples in Table 1). As in the previous studies (Desai et al., 2011, 2013; Lai et al., 2019), each triple consisted of a Lit sentence using a hand−/arm-related action verb to describe a physical action, a Met sentence utilizing the same verb figuratively to convey abstract meaning, and an Abs sentence employing an abstract verb to express the same meaning as the Met sentence. Each sentence’s agent was selected to suggest a literal or figurative/abstract reading of the verb. For Met and Abs sentences, the agent referred to an entity which could not make physical actions, and the agents of these two types of sentences in one triple were always the same. In contrast, for literal action sentences, the agent was a person. The Filler sentences featured different syntax from the experimental sentences. Table 1 shows the examples of experimental materials.

**Table 1:** Examples of experimental materials.

Lit	小偷**推**翻了桌子 (The thief *overturned* the table)	医生**打破了**杯子 (The doctor *broke* the cup)
Met	团队**推翻**了提案 (The team *overturned* the proposal)	剬司**打破**了规则 (The company *broke* the rules)
Abs	团队**反对**了提案 (The team *rejected* the proposal)	剬司**违反**了规则 (The company *violated* the rules)

To get these experimental sentences, we first selected 100 sentences from CCL corpus,^1^ The Modern Chinese Dictionary (the Commercial Press, 5th edition) and other related studies (Desai et al., 2011, 2013; Ji et al., 2020). These 100 sentences were adapted by five PhD students majoring in linguistics to fit the experimental demands and then underwent a series of pretests to exclude the influence of other variables. All participants (3 groups, 25 participants in each) in the pretests were native speakers of Chinese and did not participate in the formal ERP experiment.

The first group of subjects were invited to rate the meaningfulness, familiarity and action association of the 100 sentences by using 7-point-likert-scales (1 = strongest, 7 = weakest). Sentences whose average scores of meaningfulness and familiarity were less than 3.5 were excluded, and there was no significant difference in meaningfulness and familiarity (ps > 0.05) among the three types of experimental materials. The third pretest was an action association rating. Although we have purposefully chose the materials, it was plausible that some of these abstract verbs still had some link with physical actions. This pretest was conducted to ensure that all abstract verbs in this experiment were less associated with body actions. The mean score (sd.) of Lit sentences was 6.26 (0.41), and the mean score of Abs sentences was 2.98 (0.80), *p* < 0.001.

The second and the third group of subjects were asked to do the “subject-predicate” and “predicate-object” cloze probability tests separately. In the “subject-predicate” cloze probability test, all sentences had their subjects replaced with blanks, and participants were asked to fill in the blank with the first word that came to mind to complete the sentence. Similarly, the “predicate-object” cloze probability was tested by the third group of participants. To avoid predictive inertia for experimental materials (Schaller et al., 2017), we selected only those sentences with an average cloze probability of “subject-predicate” and “predicate-object” less than 10% as experimental materials. Finally, 23 triples were created, producing 23 sentences in each of the Lit, Met and Abs conditions. In order to avoid the strategy effect, 27 Filler sentences (used to obscure the triple construction of the stimuli) with the different sentence structure and different verbs (including both action and abstract verbs) were created. Twenty of them were nonsense sentences, and the others did make sense. All the sentences were divided into 3 blocks, and sentences in each block were arranged in pseudo-random order to avoid sequential effect. Each type of sentence was distributed as evenly as possible in every testing block.

### Research design and procedure

**Figure 1:**
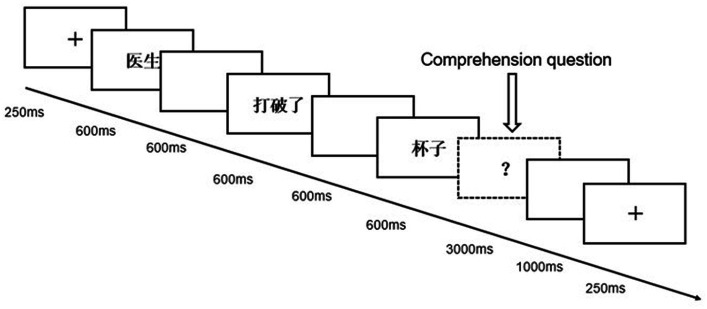
Experimental procedure.

The experimental materials were programmed with E-prime 2.0 software. The experimental materials were divided into three blocks using a Latin square design so that sentences from one triplet were presented in different blocks, each block consisted of 23 experimental sentences and 9 Filler sentences, and the sentences in the blocks were presented randomly. Before the experimental set, a practice set with 12 trials (including 4 Filler sentences) was ran first. Stimuli were presented on a 19-inch monitor with a white background and were displayed in black letters in Song typeface with 36 font size. In the practicing part, a fixation cross “+” was firstly displayed in the center of the screen for 250 ms to remind the subjects of the beginning of the experiment. The trials were then presented word-by-word, with each word being displayed for a duration of 600 ms, followed by a blank screen lasting 600 ms. After the presentation of several sentences, a randomly selected yes-no comprehension question was presented to each participant. Participants were instructed to respond with accuracy and speed by pressing the “F” and “J” keys on the keyboard. The sequence of the yes/no answer was balanced. After responding within 3,000 ms, the participants were presented with a blank screen for 2,000 ms before proceeding to the next trial. If the subject failed to respond within 3,000 ms, the question disappeared and they were also presented with a blank screen for 2,000 ms before moving on to the next trial. Please refer to Figure 1 for more detailed information about the experimental procedures.

In this study, participants were not obliged to provide explicit responses to the target verbs in order to avoid recording brain activity associated with motor response preparation and execution. At the end of the practice block, a prompt appeared to ask the participants if they have understood the whole process. Participants chose to go on or go back by pressing the key buttons. The procedure of the experimental set was the same as that of the practicing set. During the experiment, they could choose to take a rest during in-between blocks.

### Electrophysiological recording and statistic analysis

Participants were seated in a comfortable and height-adjustable chair in the Language and Brain Science Lab of Ocean University of China. They were seated at a distance of 140 cm from the computer monitor, and the resolution of the computer screen was 1,024 × 768. The behavioral data were recorded by the psychological software E-Prime 3.0.

The scalp EEGs were recorded using a 32-channel Ag-AgCl electrode cap, with a sampling rate of 1,000 Hz and a band-pass filter ranging from 0.01 to 100 Hz. Offline referencing was performed for all electrodes to the mean of two mastoids. Vertical eye movements were monitored through a supra-to sub-orbital bipolar montage. An EEG amplifier was used to amplify the EEG data. The impedance of the electrodes was consistently maintained below 5kΩ throughout the whole experiment.

**Figure 2:**
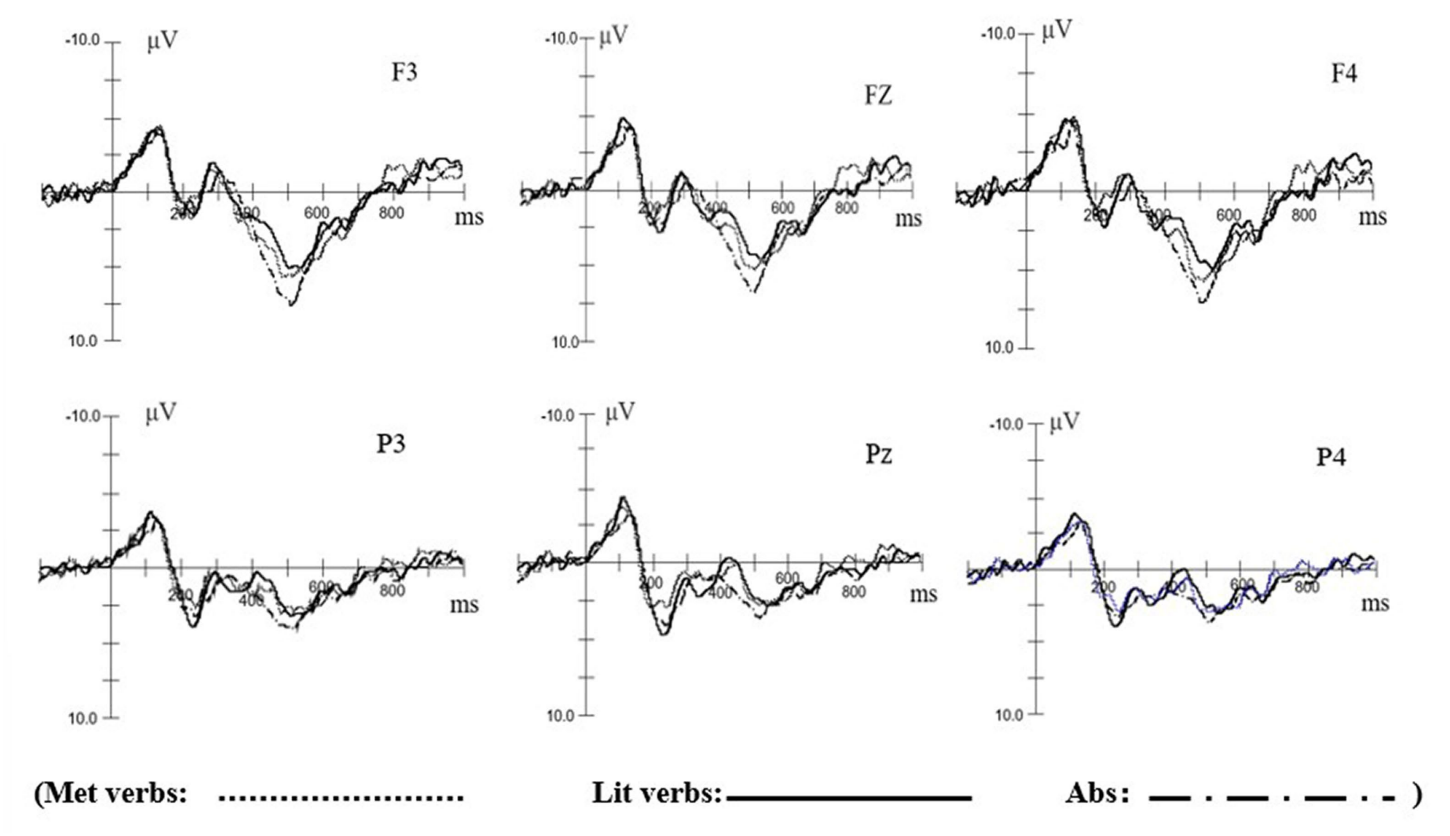
Averaged ERP responses for Lit, Met, and Abs verbs.

Curry 8 software was used to process the acquired ERP data in the off-line processing. The main steps included baseline correction, filtering, artificial reduction (threshold and bad block), epoch and averaging. When segments contained signals exceeding ±100 μV, they were rejected. After artificial reduction, only 23 out of 25 participants’ data entered into SPSS 20.0 for further statistical analysis. The epochs ranged from 200 to 1,000 ms and were time-locked to the onset of words, and a baseline correction was applied from-200 to 0 ms prior to the target word onset. Offline calculations were then performed on the average waveforms. Figure 2 demonstrates the averaged ERP responses at electrodes for Lit verbs, Met verbs, and Abs verbs. The horizontal axis shows the time-course, and each tick represents 100 ms. The vertical axis shows the amplitude, and each tick represents 2.5 μV.

After the exaction of the EEG data, 23 participants’ data entered into SPSS 20.0 for further statistical analysis. Statistical analyses were carried out in the time window of 200–500 ms, 200–300 ms, and 300–500 ms separately. For the N400 component, the averaged amplitudes of 200–500 ms were analyzed using repeated-Measures ANOVAs with the factors of condition (Lit, Met, Abs) and region of interest (frontal: F3/F4/Fz, posterior: P3/P4/Pz). Then, we separated the time window into two sections (200–300 ms and 300–500 ms) based on the visual inspection of the grand averaged data in order to better capture the temporal dynamics of the brain activity and determine whether the Chinese configuration and stroke would influence the concreteness N400 effect, as the time differences may reflect differential underlying neural mechanisms (Lai et al., 2019). The premise of this approach is the assumption of sphericity, which means that all variances and all correlations of the pairs of repeated measurements must be equal. If this assumption fails, Greenhouse–Geisser sphericity correction would be conducted. The present study only reported the corrected *p* values, and the degrees of freedom are original ones.

## Results

### Behavioral results

Behavioral data in this experiment only included accuracy to assess whether the subjects concentrate on the experimental materials or not during the whole process. SPSS 20.0 was used to calculate the accuracy of the subjects, and the results showed that the mean accuracy rate of all participants was 87.5%, the lowest was 75%, and the highest was 98.78%. The results of behavioral data indicated that the participants demonstrated attentiveness toward the experimental sentences throughout the whole experiment.

### ERP results

According to the previous research on metaphor processing (Lai et al., 2009; Bambini et al., 2016; Lai et al., 2019) and visual inspection of the averaged ERP responses, we selected the 200–500 ms time window for the analysis of the N400 effect elicited by predicate verb. We then analyzed the time window of 200–300 ms and 300–500 ms separately to further clarify the underlying neural mechanisms.

#### N400 (200–500 ms)

Mean amplitudes for the three conditions from 200 to 500 ms were extracted and analyzed using Repeated-Measures ANOVA of 3 condition (Lit, Met, Abs) × 2 region of interest (frontal, posterior). The results revealed significant main effect of condition for both frontal (*F* (1, 22) = 3.847, *p* = 0.029) and posterior (*F* (1, 22) = 4.882, *p* = 0.023) regions. Further analysis indicated that at the frontal region, Lit verbs elicited a significantly more negative mean amplitude over Abs verbs (*F* (1, 22) = 3.432, *p* = 0.033), Met verb evoked a significantly more negative mean amplitude over Abs verb (*F* (1, 22) = 2.849, *p* = 0.043), and at the posterior region, Met verbs elicited a significantly more negative mean amplitude over Lit verbs at the posterior location (*F* (1, 22) = 3.378, *p* = 0.037).

#### 200–300 ms

Mean amplitudes for the three conditions from 200 to 300 ms were extracted and analyzed using Repeated-Measures ANOVA of 3 condition (Lit, Met, Abs) × 2 region of interest (frontal, posterior). The results showed a significant main effect of condition at the frontal region (*F* (1, 22) = 3.052, *p* = 0.041), and further analyses showed that Lit verbs elicited a significantly more negative mean amplitude over Abs verbs (*F* (1, 22) = 3.130, *p* = 0.041), and Met verb evoked a significantly more negative mean amplitude over Abs verb (*F* (1, 22) = 2.929, *p* = 0.045).

#### 300–500 ms

Mean amplitudes for the three conditions from 300 to 500 ms were extracted and analyzed using Repeated-Measures ANOVA of 3 condition (Lit, Met, Abs) × 2 region of interest (frontal, posterior). The results showed a significant main effect of condition at the frontal brain region (F (1, 22) = 3.634, *p* = 0.032), and further analyses showed that the mean amplitude elicited by Lit verbs was more negative than Abs verbs (*F* (1, 22) = 3.584, *p* = 0.030), and the mean amplitude elicited by Met verbs was more negative than Abs verbs (*F* (1, 22) = 3.846, *p* = 0.029). A significant main effect of condition at the posterior region was also found in this time window, and further analysis indicated that Met verbs elicited more negative mean amplitude than Lit verbs (*F* (1, 22) = 4.021, *p* = 0.013).

## Discussion

To explore the processing mechanism of the metaphorical use of action verbs for Chinese native speakers, the present study examined the event-related brain response to visually presented Met verbs. To be more specific, this study compares the mean amplitudes activated by the Met verbs with those activated by the Lit verbs and the Abs verbs. Given the lack of clear establishment regarding ERP correlates of sensory-motor recruitment, we employed the concreteness N400 effect to approximate the data. The concreteness N400 effect has been widely investigated and proved to be the indicator for the comprehension of concrete semantic (Zhang et al., 2006; Adorni and Proverbio, 2012; Barber et al., 2013; Lai et al., 2019; Chen et al., 2022; Xia and Peng, 2022). Therefore, it is feasible to estimate the processing mechanism of Met verbs through the utilization of concreteness N400 (Lai et al., 2009). The findings revealed that the Met verbs evoked a significantly larger N400 response at frontal brain region compared to the Abs verbs at 200–500 ms time window, while the Met verbs elicited a notably greater N400 amplitude specifically at the posterior brain region in comparison to the Lit verbs at 300–500 ms time window.

According to ERP data, the ERP components of N400 is the main indexes to explore the mental representation of the metaphorical meaning of Chinese action verbs. The present study found a concreteness N400 effect when comparing Lit verb with Abs verb, which is largely consistent with the previous studies (Hauk et al., 2006; Zhang et al., 2006; Kim and Lai, 2012; Barber et al., 2013; Vukovic et al., 2015; Lai et al., 2019). However, its activation time is slightly different from some of the previous studies. In terms of timing, the concreteness N400 effect begins at 200 ms after the onset of the Lit verb in the present study, which is earlier than the findings of Zhang et al. (2006), Kanske and Kotz (2007), Adorni and Proverbio (2012), and Barber et al. (2013). The reason may be that the focuses of the present study and other studies are different. The materials in the present study were embedded in sentence contexts whereas others focused on single words. This reason has also been mentioned in Lai et al. (2019) which also found a concreteness N400 effect at the 200–500 ms time window, and its materials were also sentences. According to Lai et al. (2019), even if it was only a few words preceding the target verbs, sentential context may have induced the pre-activation of the verbs meaning to some extent, thereby leading to an earlier time-window. By examining the influence of processing action verbs in the corresponding body movements, Boulenger et al. (2006) found that the interference effects could be observed within 200 ms after verb onset. The results were interpreted by the authors as reflecting the early involvement of the cortical motor regions in the processing of action verbs.

In terms of scalp distribution, the present study found that Lit verbs elicited a significantly more negative mean amplitude at the frontal location, which is consistent with the results of Bardolph and Coulson (2014), Dalla Volta et al. (2014), and Lai et al. (2019). There studies linked the negative ERP component in the time window of 200–300 ms at the frontal region to embodiment. For instance, Bardolph and Coulson (2014) examined the embodiment of processing words associated with spatial attributes (e.g., ascend, descend) and found a frontally distributed negative ERP component from 200 to 300 ms after word onset. Dalla Volta et al. (2014) found a concreteness N400 effect at the frontal brain region when comparing concrete action verbs with abstract verbs, and they performed source estimation for this concreteness effect and detected a cortical cluster in the sensorimotor region near the rolandic fissure. Furthermore, it was observed that the activation of hand-related verbs originates from the precentral gyrus. Lai et al. (2019) explored the processing mechanism of action verbs in literal and metaphorical situation. They found a significant concreteness N400 effect (comparing Lit verb and Abs verb) in the frontal brain region and ascribed it to the activation of the sensorimotor system. The findings of the present study are substantially consistent with the results of these research.

We also found a N400 effect (200–500 ms after word onset) at the frontal brain region when comparing Met verb with Abs verb, which is the main focus of the present study. The primary inquiry of this study pertained to the existence of a concreteness effect during the cognitive processing of action verbs within metaphorical contexts. This N400 effect seems to indicate that the literal meaning was accessed early (starting at 200 ms post verb onset) during the comprehension of Met verb and that the sensorimotor system was activated in this process according to the above discussion. However, the results do not mean that we support the “literal-first” view of metaphor processing, as the frontal N400 effect elicited by Met verbs was similar to that caused by Lit verbs (from 200 to 500 ms post verb onset). If this early N400 effect is attributable to the access of the literal meaning of Met verbs which is suppressed as soon as the metaphor is identified (Reilly et al., 2019), it should dissipate within 300 ms after verb onset (Lai et al., 2019). Therefore, the present study thus provides empirical evidence supporting the assertion that the metaphorical usage of a verb preserves its fundamental semantic component (Torreano et al., 2005; Cacciari et al., 2011). Another reason that is worth noticing is the special physical features of Chinese characters. In the present study, most of the action verbs are with the radical “扌” (e.g., 拍/pat, 摸/touch, 抓/grasp) which is a variant of a Chinese character “手” (“hand” in English). Therefore, the radical “扌” is closely related with hand actions, which might provide semantic clues to subjects and then cause the activation of the sensorimotor systems before the semantic access of the action verbs (starting 200 ms after verb onset). In other words, there may be an automatic activation of the sensory-motor systems when participants see characters with “扌” as their radicals, thus causing similar motor activation during the processing of action verbs both in metaphorical and literal sentences. Future studies could explore whether action verbs with the radical “扌” and those without the radical cause different N400 effects. In brief, these results are mostly in line with previous findings about the representation of the metaphorical meaning of action verbs. The activation of frontal area in the early processing stage of the metaphorical meaning of action verbs indicates that there may be engagement of sensory-motor system during Met verbs comprehension.

For Met and Lit verbs, there are no significant difference between them at the frontal brain area in the time window of 200–500 ms, but Met verb elicited a significantly more negative mean amplitude over Lit at the posterior location in the 300–500 ms time window, which is mostly compatible with the N400 effect (i.e., the classic N400 effect) documented in previous ERP studies. These results seem to indicate that the processing of Met verbs and Lit verbs elicited similar activation of the frontal area. But at posterior electrode sites, the Met verbs elicited a larger N400 than Lit verbs, which is more likely consistent with the classic N400. The classic N400 effects are typically interpreted as reflecting the relative difficulty of lexical/semantic integration processes, whereby a word’s fit with the context is inversely related to the magnitude of the N400 amplitude (Kutas and Federmeier, 2011). In the present study, the predicates of Met sentences and Lit sentences are all concrete action verbs literally, but for Met sentences, abstract meanings are expressed by the analogy to concrete concepts. The process of aligning the metaphorical interpretation of the verb with sentence context takes place in this processing stage (Bowdle and Gentner, 2005; Tzuyin Lai and Curran, 2013). Therefore, processing Met verbs may take more brain energy than the processing of Lit verbs in homing in the sentence contexts.

## Conclusion

In the present study, we examined the concreteness N400 effect in response to Chinese Met verb comprehension within the framework of embodied language theory. We found that Lit verb and Met verb elicited similar concreteness N400 effect at the frontal brain region when compared with Abs verb. However, these findings do not directly demonstrate the activation of the sensorimotor cortex during the processing of Met verb. We merely indicate that Met verbs are comprehended similarly to Lit verbs in terms of the frontally distributed concreteness, which may be interpreted as that the comprehension of the Met verbs is based on the concrete action semantics. One limitation of the present experiment is that the scope of this study is limited to Chinese action metaphor sentences with a subject-predicate-object structure, and does not encompass the analysis of Chinese action metaphors at other linguistic levels, such as phrases and texts. Therefore, the findings presented in this study are exploratory in nature and require further validation. Future studies could explore the impact of various contextual factors like sentence structure, surrounding words, or even discourse coherence on Met verb comprehension.

## Data availability statement

The original contributions presented in the study are included in the article/supplementary material, further inquiries can be directed to the corresponding authors.

## Ethics statement

The studies involving humans were approved by Ethics Review Committee of the School of Foreign Languages, OUC. The studies were conducted in accordance with the local legislation and institutional requirements. The participants provided their written informed consent to participate in this study. Written informed consent was obtained from the individual(s) for the publication of any potentially identifiable images or data included in this article.

## Author contributions

YZ: Formal analysis, Methodology, Software, Supervision, Writing – original draft, Writing – review & editing. SC: Supervision, Writing – original draft, Writing – review & editing. YP: Formal analysis, Validation, Writing – original draft, Writing – review & editing. XY: Resources, Writing – original draft, Writing – review & editing.

## Footnotes

^1^ http://ccl.pku.edu.cn:8080/ccl:corpus/index.jsp
